# Aortic Valve Dysfunction and Aortopathy Based on the Presence of Raphe in Patients with Bicuspid Aortic Valve Disease

**DOI:** 10.3390/jcdd10090372

**Published:** 2023-08-31

**Authors:** Yu Zhang, Bo Hwa Choi, Hyun Keun Chee, Jun Seok Kim, Sung Min Ko

**Affiliations:** 1 Department of Radiology, Yuhuangding Hospital, Yantai 264008, China; jang__ok@naver.com; 2 Department of Radiology, Wonju Severance Christian Hospital, Yonsei University Wonju College of Medicine, Wonju 22070, Republic of Korea; 3 Department of Radiology, National Cancer Center, Goyang 10408, Republic of Korea; iawy82@gmail.com; 4 Department of Cardiovascular Surgery, Konkuk University Medical Center, Konkuk University School of Medicine, Seoul 05030, Republic of Korea; 20050711@kuh.ac.kr (H.K.C.); drheart@kuh.ac.kr (J.S.K.)

**Keywords:** bicuspid aortic valve, computed tomography, aortic regurgitation, aortic stenosis, aortopathy

## Abstract

(1) Background: To identify the association between the presence or absence of a raphe and aortic valve dysfunction, as well as the presence of aortopathy in patients with a bicuspid aortic valve (BAV); (2) Methods: This retrospective study enrolled 312 participants (mean (SD) age, 52.7 (14.3) years; 227 men (72.8%)) with BAV. The BAVs were divided into those with the presence (raphe+) or absence (raphe−) of a raphe. Valvular function was classified as normal, aortic regurgitation (AR), or aortic stenosis (AS) using TTE. The pattern of BAV aortopathy was determined by the presence of dilatation at the sinus of Valsalva and the middle ascending aorta using CCT; (3) Results: BAVs with raphe+ had a higher prevalence of AR (148 (79.5%) vs. 48 (37.8%), *p* < 0.001), but a lower prevalence of AS (90 (48.6%) vs. 99 (78.0%), *p* < 0.001) compared with those with raphe−. The types of BAV aortopathy were significantly different (*p* = 0.021) according to those with BAV–raphe+ and BAV–raphe−; (4) Conclusions: The presence of a raphe was significantly associated with a higher prevalence of AR, but a lower prevalence of AS and combined dilatation of the aortic root and middle ascending aorta. The presence of a raphe was an independent determinant of AR.

## 1. Introduction

A bicuspid aortic valve (BAV) is the most common congenital heart disease, affecting 0.5% to 2% of the general population [1]. Although a BAV can be an isolated disorder, it is often associated with serious valvular disorders, including aortic stenosis (AS) and aortic regurgitation (AR), as well as aortic wall abnormalities such as aortopathy, aneurysm, and dissection. BAVs can anatomically vary in terms of the number of commissures, cusps, and raphes. The association between variability of the phenotype, and valvular and aortic morbidity has been discussed, but remains controversial in many studies [2]. An accurate assessment of the BAV morphology, particularly the presence of a raphe and the burden of calcification, as well as the aorta morphology, are clinically important for selecting an appropriate treatment and minimizing complications [3].

Transthoracic echocardiography (TTE) is the standard diagnostic imaging modality for evaluating BAV patients [4]. However, the operator’s skill, poor acoustic window, extensive calcifications in the valve, and patient body habitus may make the diagnostic accuracy of TTE challenging [4]. Retrospective electrocardiography (ECG)-gated multidetector computed tomography (MDCT) is considered to be more accurate than TTE for evaluating aortic valve morphology and the associated aortopathy by providing morphological information and functional cine imaging [5]. Therefore, when TTE fails or is inconclusive, cardiac computed tomography (CCT) is considered an alternative diagnostic imaging tool for evaluation of the aortic valve [5].

To the best of our knowledge, most studies on the association between BAV morphology and patterns of valvular dysfunction, as well as bicuspid aortopathy, were based on the position and number of cusps as well as commissures [6,7], which were performed mostly using TTE in a Western population [8]. Therefore, this study aimed to determine whether the presence or absence of a raphe is associated with various degrees of valvular dysfunction and different types of aortopathy using TTE and CCT in a Korean population, and identify whether the presence or absence of a raphe is a determinant of valvular dysfunction.

## 2. Materials and Methods

This retrospective, single-institution study was approved by the Institutional Review Board and Local Ethics Committee (KUMC-2020-02-014). The requirement for written informed consent was waived.

### 2.1. Patients

This study cohort included 312 BAV patients who underwent both CCT and TTE within four weeks from January 2008 to April 2019, without an intervening change in clinical status or cardiovascular events. The baseline clinical characteristics, cardiovascular risk factors, and cardiac evaluation results were collected from medical and radiological records. CCT was performed for 2 different reasons. First, CCT was performed to evaluate coronary artery disease in patients with chest pain. Because the BAV was incidentally diagnosed via CCT, TTE was performed after CCT. Second, CCT was performed as a planned imaging study after echocardiography in patients with suspected or known aortic valvular heart disease. The purpose of CCT in these patients was to evaluate preoperative coronary artery anatomy and stenosis, aortic valve morphology, aortic valve cusp calcification, and ascending aortic dimensions.

### 2.2. CT Imaging Protocols

A dual-source CT scanner (Somatom Definition; Siemens Medical Solutions, Forchheim, Germany) was used at our institution. To acquire good-quality cardiac imaging, patients with a pre-scan heart rate (HR) of >65 beats per minute (bpm) without contraindications for beta-blockers were administered 50–100 mg of metoprolol orally for 1 h to control the HR. All patients received 0.6 mg of nitroglycerin sublingually 1 min before examination to dilate the coronary arteries.

CCT scans were performed from 2 cm above the carina to the diaphragm, without including the entire aortic arch. The detector collimation was 2320.6 mm, with a slice acquisition of 2640.6 mm, the gantry rotation time was 330 ms, the pitch of 0.20–0.43 was adapted to the HR, and the tube voltages were 100 or 120 kV. A tube current–time product of 100–140 mAs per rotation was used for calcium scoring and 100–280 mAs per rotation for CCT. A non-enhanced ECG-gated CCT scan, prospectively triggered at 75% of the R–R interval, measured the coronary artery and aortic valve calcium scores. Except for patients with mean HRs > 80 bpm or those with arrhythmia, ECG-based tube current modulation was used for CCT. A full-dose window of 20–70% of the cardiac cycle was used in patients with a HR ≤ 80 bpm.

The contrast agent was administered using the bolus-tracking technique. For all CT examinations, a dual-head power injector (Stellant D; Medrad, Indianola, PA, USA) was used to administer the three-phase bolus at a rate of 4.5 mL/s. First, 70–80 mL of iopromide (Ultravist 370^®^; Bayer Healthcare, Berlin, Germany) was administered. Thereafter, 45 mL of a 70-to-30% blend of a contrast medium and saline were added. Finally, 45 mL of saline was administered.

### 2.3. CT Image Reconstruction and Analysis

To assess BAV morphology and function, images were reconstructed parallel to the aortic valve plane with retrospective ECG gating every 10% of the cardiac cycle, from 0% to 90% of the R–R interval. The images were reconstructed with a slice thickness of 0.75 mm and an increment of 0.4 mm. Non-enhanced CCT images were reconstructed with a section thickness of 3 mm and a reconstruction interval of 1.5 mm to quantify the coronary artery and aortic valve calcium scores. Oblique coronal and oblique sagittal planes of the aortic valve during the entire cardiac cycle were reconstructed to measure the morphological and functional aspects of the BAV, and double-oblique transverse images were reconstructed to measure the dimensions of the ascending aorta. Once the reconstruction was completed, all datasets were transferred to a post-processing workstation (Vitrea 2, Vital Images, Plymouth, MN, USA) to evaluate the phenotype and function of the aortic valve and measure the ascending aortic dimensions.

**Figure 1:**
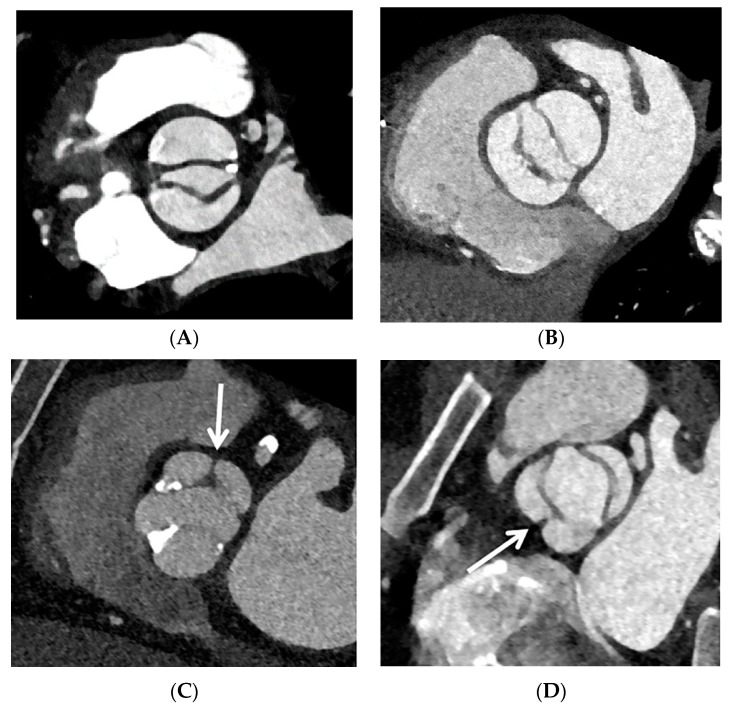
Bicuspid aortic valve classification: valve type according to the absence (**A**,**B**) or presence (arrow, **C**,**D**) of a raphe on cardiac CT in double-oblique short-axis plane in the systole.

**Figure 2:**
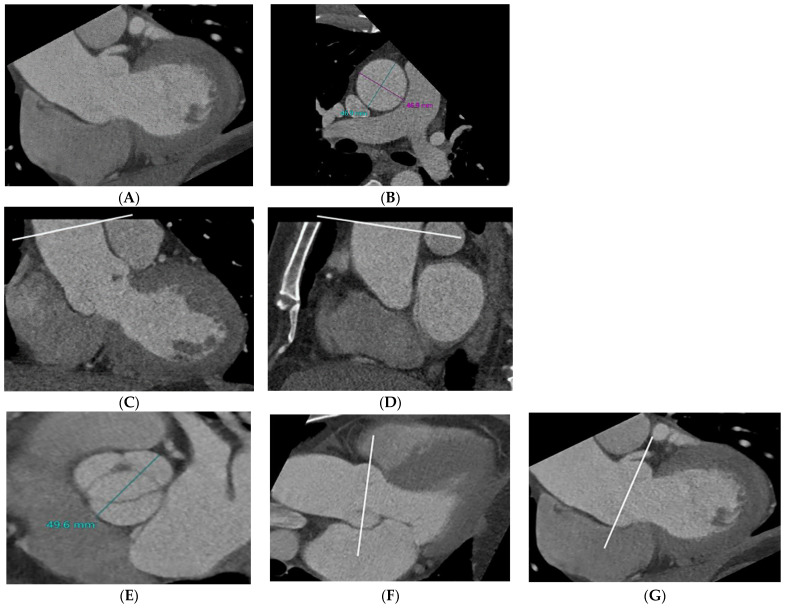
Example of the measurement of the aortic dimensions at different locations. Oblique sagittal reformatted CT image shows the sinus of Valsalva and tubular portion of the ascending aorta at mid-systole (**A**). The maximum dimension of the tubular portion of the ascending aorta (white line) was measured in a double-oblique transverse view (**B**) obtained perpendicular to the aortic lumen (**C**,**D**) because the axial measurement exceeded the true measurement perpendicular to the long axis of the ascending aorta. The maximal aortic root dimension (white line) was measured sinus-to-sinus in a bicuspid aortic valve with a raphe (**E**) in a double oblique transverse view obtained perpendicular to the aortic sinus of Valsalva (**F**,**G**).

**Figure 3:**
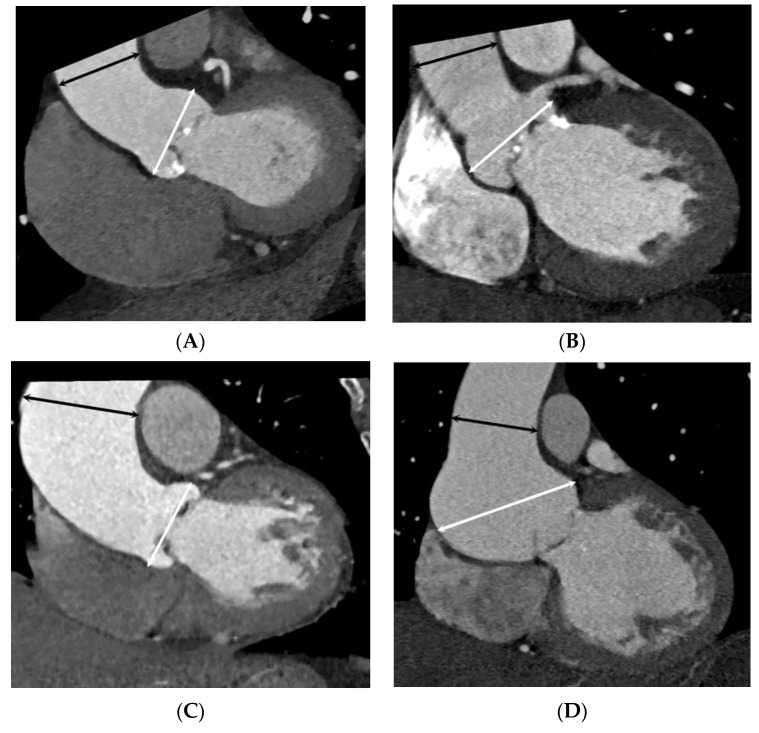
Diameters of the ascending aorta measured via cardiac computed tomography at two different levels (sinus of Valsalva [white arrow] and tubular portion [black arrow]) at mid-to-end systole. The ascending aorta was assigned to one of four main anatomical types according to the segment of the vessel exclusively or predominantly involved in dilatation based on the indexed maximal aorta diameter of exceeding 21 mm/m^2^ of body surface area: (**A**) normal; (**B**) type 1, root dilatation; (**C**) type 2, ascending aorta dilatation; (**D**) type 3, combined dilatation of the aortic root and middle ascending aorta.

All CCT images were reviewed by a consensus of two radiologists with 16 and 5 years of experience, respectively, who were blinded to the clinical and surgical data. A BAV was defined as the presence of two cusps and commissures in both the systole and diastole. The following morphological variables were assessed: (1) the presence or absence of a raphe and (2) the ascending aortic diameter. The term “raphe” defines the conjoined or “fused” area of two adjacent undeveloped leaflets that turn into a malformed commissure between both leaflets [9]. BAV morphology was classified as raphe+ or raphe− according to the presence or absence of a raphe, respectively (Figure 1) [10]. The dimensions of the ascending aorta were measured at the sinus of Valsalva and tubular portion. Double-oblique coronal images of the ascending aorta were reconstructed at 10% or 20% of the cardiac cycle (early to mid-systole) to measure the tubular portion of the ascending aorta dimensions. The measurement of the maximum dimension of the aortic sinuses of Valsalva was performed using a double-oblique transverse view of the aortic root at 10% or 20% of the cardiac cycle (Figure 2). Aortopathy refers to the progressive dilatation of the ascending aorta and is defined as an indexed maximal aortic diameter exceeding 21 mm/m^2^ of the body surface area (BSA) [11]. Ascending aortic dilation configurations were slightly modified from the Fazel classification, which was assigned to four types depending on whether the segment of the vessel was exclusively or predominantly involved in dilatation: normal aorta; type 1, isolated dilation of the ascending aorta root at the level of the sinus of Valsalva [12]; type 2, middle ascending dilatation at the level of the tubular ascending portion; and type 3, combined dilatation of the aortic root and mid-ascending aorta (Figure 3) [12]. Aortic aneurysms are defined as those with dimensions greater than 50 mm. The association between the presence of a raphe, and the risk of aortic valve dysfunction and aortopathy was also analyzed.

### 2.4. Echocardiographic Evaluation

All patients underwent two-dimensional (2D) TTE using a Vivid 7 device (GE Healthcare, Wauwatosa, WI, USA) and an Acuson Sequoia C512 apparatus (Siemens, Erlangen, Germany) with 2.5–3.5 MHz imaging transducers. Aortic valve morphology and the severity of aortic valve dysfunction were reviewed by cardiologists. Aortic valve function was recorded as normal, AR, AS, and combined AS and AR (ASR) using TTE [13], and their severity was graded as none, mild, moderate, or severe based on current recommendations [14]. Left ventricular functional parameters were measured on 2D TTE using a modified Simpson’s method.

### 2.5. Statistical Analyses

For descriptive statistical analysis, continuous variables are presented as the mean ± standard deviation (SD), and categorical variables are presented as numbers or percentages. The chi-square or Fisher’s exact test for categorical data and the unpaired two-tailed t-test for continuous data examined the significant differences between BAV patients with raphe+ vs. raphe−. The independent association between the presence of a raphe and the severity and type of aortic valve dysfunction was evaluated using binary logistic regression analysis, with AR and AS being the dependent variables, and sex, age, cardiovascular risk factors, and the presence of a raphe the independent variables. Statistical significance was set at *p* < 0.05. All data analyses were performed using Statistical Analysis System (SAS) version 9.4 (SAS Institute Inc., Cary, NC, USA).

## 3. Results

**Figure 4:**
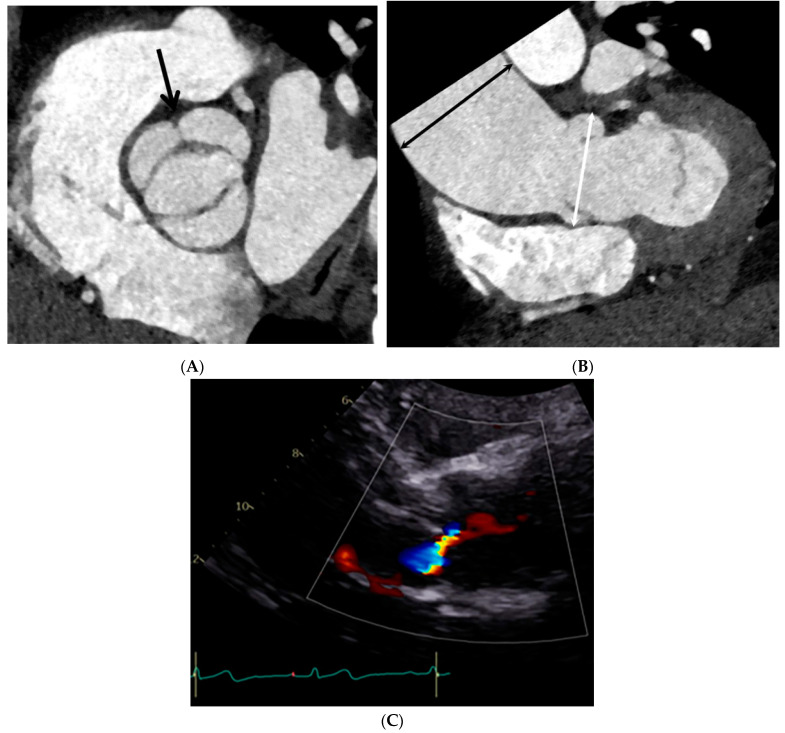
Bicuspid aortic valve (BAV) with a raphe with ascending aorta root dilatation in a 48-year-old male. (**A**) Double-oblique CT reconstruction parallel to the aortic valve during systole demonstrates a BAV with fusion of the right and left coronary cusps. (**B**) Double-oblique coronal CT reconstruction through the left ventricular outflow tract during systole shows the diameters of the sinuses of Valsalva (white arrow) at 40.8 mm (size index 2.02 cm/m^2^) mm and the mid-ascending aorta (black arrow) at 53.6 mm (size index 2.65 cm/m^2^), which were measured at mid-systole. (**C**) Color Doppler echocardiogram shows a jet regurgitating into the left ventricle. Aortic regurgitation was considered mild.

**Figure 5:**
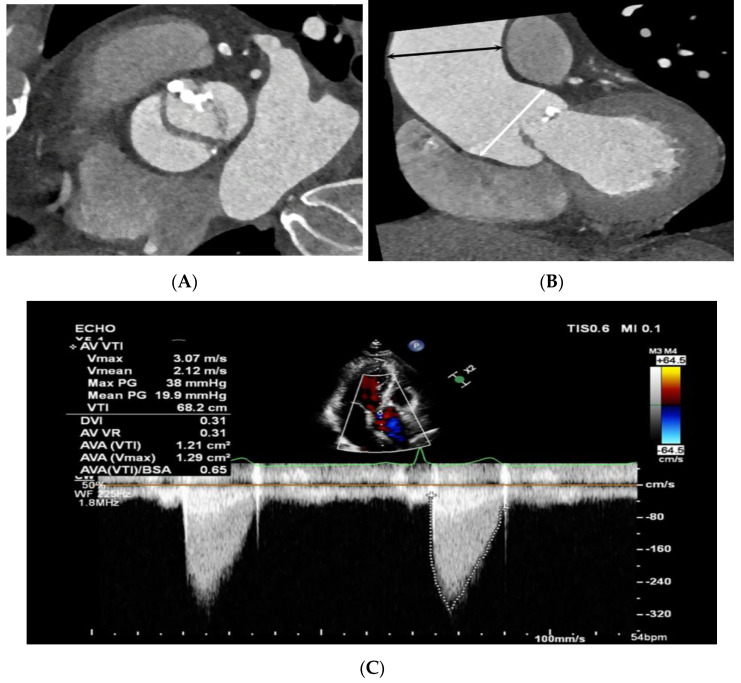
Bicuspid aortic valve (BAV) without a raphe and combined aortic root, as well as mid-ascending aorta in a 64-year-old male. (**A**) Double-oblique CT reconstruction parallel to the aortic valve demonstrates thickened, calcified BAV cusps without a raphe. (**B**) Diameters of the sinuses of Valsalva (white arrow) at 43.5 mm (size index 2.24 cm/m^2^) and the mid-ascending aorta (black arrow) at 46.4 mm (size index 2.48 cm/m^2^), which were measured at mid-systole. (**C**) Continuous-wave Doppler recording of the aortic stenosis jet from an apical approach shows a maximum velocity of 3.07 m/sec. The continuity equation of the aortic valve area was 1.2 cm^2^, corresponding to moderate aortic stenosis.

A total of 312 BAV patients (mean [SD] age, 52.7 [14.3] years) were identified and enrolled in this study. No patient had two raphes. Of these, 227 were male (72.8%) and 185 (53.9%) were BAV–raphe+. Table 1 summarizes the differences between the BAV–raphe+ and BAV–raphe− groups. The BAV–raphe+ group had more males (156 [84.3%] vs. 71 [55.9%]; *p* < 0.001), a larger BSA (1.8 ± 0.2 vs. 1.7 ± 0.2; *p* = 0.001), and were more smokers (mean [SD], 80 [43.2%] vs. 37 [29.1%]; *p* = 0.011), compared with the BAV-raphe− group. A larger aortic annulus, a larger sinus of Valsalva diameter, and smaller tubular portion were more often observed in BAV–raphe+ patients (29.8 ± 4.3 mm vs. 26.7 ± 3.8 mm, 40.2 ± 6.4 mm vs. 38.7 ± 5.6 mm, 42.1 ± 7 mm vs. 44.3 ± 8.3 mm, respectively; *p* < 0.05). The difference persisted only in the tubular portion after indexing the proximal aortic diameter for BSA (24.2 ± 5 mm vs. 26.6 ± 5.6 mm; *p* < 0.001). Although the morphology of the BAV was not associated with aortopathy, the distribution of various aorta types was significantly different between the two groups (*p* = 0.021). In particular, isolated dilation of the aortic root (type 1) was the predominant form for BAV patients with raphe+ (13.5% [25 of 185] vs. 6.3% [8 of 127] in raphe−) (Figure 4), but the prevalence of combined dilation of the ascending aorta and aortic root (type 3) was most common in BAV patients with raphe− (72.4% [92 of 127] in raphe− vs. 55.7% [103 of 185] in raphe+) (Table 2, Figure 5). The presence of a raphe was significantly correlated with the pattern of valvular dysfunction (*p* < 0.0001). No AS and severe AR were more common in BAV patients with raphe+, whereas severe AS and no AR were the more common patterns in BAV patients with raphe−. Dominant AR (defined as moderate-to-severe AR with no or mild AS) was present in 73 patients with raphe+ (39.5%) and 8 patients with raphe− (6.3%); dominant AS (defined as moderate-to-severe AS with no or mild AR) was present in 70 patients with raphe+ (37.8%) and 82 patients with raphe− (64.6%); balanced ASR (defined as moderate-to-severe combined AS and AR) was present in 10 patients with raphe+ (5.4%) and 9 patients with raphe− (7.1%) (Table 1).

During multivariate analysis, female sex (OR: 0.2, 95% CI: 0.1–0.4, *p* < 0.001), aortic valve calcium score (OR: 1, 95% CI: 1–1, *p* < 0.001), and end-diastole volume (EDV) (OR: 1, 95% CI: 1–1, *p* = 0.021) remained independently associated with AS. The presence of a raphe was significantly associated with AS following the simple logistic regression analysis (*p* < 0.001), but not in the multiple logistic regression analysis (*p* > 0.5). In contrast, younger age (OR: 1, 95% CI: 0.9–1, *p* = 0.003), EDV (OR: 1.1, 95% CI: 1.0–1.1, *p* < 0.001), end-systole volume (ESV) (OR: 1, 95% CI: 0.9–1, *p* = 0.025), and the presence of a raphe (OR: 3.7, 95% CI: 1.4–9.6, *p* = 0.007) remained independently associated with AR following both the simple and multivariable analyses (Table 3).

## 4. Discussion

In this study, the presence of a raphe in BAV patients was relatively lower than that in a previous Western study [15], and was significantly associated with a higher prevalence of AR and isolated dilatation of the aortic root and middle ascending aorta. In addition, the presence of a raphe was an independent determinant of AR. The absence of a raphe was associated with a high prevalence of AS and combined dilatation of the aortic root and middle ascending aorta.

Kong et al. [15] demonstrated that the presence of a raphe is of clinical and prognostic importance and is significantly correlated with valve dysfunction and aortic dilation. Most studies have focused on specific BAV morphology according to the orientation of leaflet fusion and the number of commissures [1,15]; however, only a few studies have focused on the presence of a raphe.

In a Western population, a raphe has been described in almost 90% of BAV patients [15]. In this study, the prevalence of BAV with a raphe was only 60%, and interestingly, the absence of a raphe was relatively more often observed than in other studies [15,16]. A BAV in Korean patients with a raphe was 52%, as indicated by Kang et al. [16]. The hypothesis that the distribution of BAV morphologies differs between Korean and Western populations could be considered for the explanation of differences in the study results [17].

Clinically, the association between the presence of a raphe and aortic valve dysfunction is more relevant [15]. However, few studies [2,18] have been conducted, particularly in Korea. A previous study showed that the prevalence of AS (44% vs. 56%) and AR (19% vs. 21%) was similar in BAV patients with raphe+ and raphe− [16,19]. However, some studies have indicated that the presence or absence of a raphe is significantly associated with the type of valvular dysfunction [15]. In this study, AR was more common in BAV patients with raphe+ and AS was more common in raphe−, the dominant AR was more common in BAV patients with raphe+, and the dominant AS was more common in raphe−. This result was confirmed in a previous study and is consistent with the study conducted by Lee et al. in Korea [20]. A raphe is an avascular fibrous mass of connective tissue that protrudes from the aortic surface. Significant redundancy of a conjoined leaflet-associated prolapse may lead to AR, whereas relatively little redundancy of valve margins with restricted motion tends to lead to AS [20]. The reasons for the discrepancies with Seviers et al. [16] may be due to the type of patient and regional (ethnic) differences included in this study. In addition, severe AR was significantly higher among BAV patients with raphe+, followed by those with moderate AR. A previous study [21] indicated that degenerative aortic valve disease occurs earlier in BAV patients than in patients with tricuspid aortic valves. Valvular motional fatigue and blood abnormalities with aging can cause damage to collagen fibers and calcification, as well as fiber thickening, and calcification progressing along the raphe can form a small coaptation defect, which causes AR.

The findings in this study are similar to those of previous studies, which showed that the female sex was significantly associated with BAV patients with AS, and AR was more common in the early period of a BAV patient’s life than AS [20]. Moreover, we demonstrated that BAV with raphe+ occurs more frequently in men than in women, whereas Kong et al. [15,16] indicated no such relationship. The small proportion of female patients and inter-ethnic differences may account for this discrepancy in the results.

Progressive dilatation of the aorta may cause aortopathy, which is a risk factor associated with an increased risk of catastrophic aortic prognosis (such as dissection and rupture) [22]. However, the relationship between BAV morphology, and the presence or absence of raphe, as well as aortopathy, remains unclear. A larger multicenter, collaborative BAV registry showed no difference in the prevalence of aortopathy between BAV–raphe+ and BAV–raphe− patients [15]. In this study, the relationship between a raphe and aortopathy was statistically significant. Genetic and hemodynamic factors are considered the underlying mechanisms of aortopathy in BAV patients [23]. The four-dimensional flow cardiac magnetic resonance imaging confirmed that diverse BAV fusion patterns were associated with the direction of the post-valvular blood jet, flow displacement, flow angles, and regional wall shear stress, which correspond to the aortic wall and jet impingement positions, resulting in the dilation of different planes [2]. However, no relationship between rotational flow and wall shear stress was found between BAVs with or without a raphe. Further studies should be conducted to identify these factors. In addition, aortic dimensions in this study were measured via cardiac CT, whereas the study measured TTE when the TTE measurement of the ascending aortic dimensions, especially the aortic root, was lower than that measured via ECG-gated CT angiography [24], and phenotypic classification was impossible in up to 20% of patients with a BAV using TTE alone [10,16].

This study had several limitations. First, the study was conducted in a single, tertiary medical center (institution) and was retrospective in design. Second, the enrolled population was heterogeneous due to the different reasons for CCT examination. Accordingly, our CCT protocol did not scan the distal ascending aorta and aortic arch because the coronary arteries and/or aortic valve were the main sites of concern. Third, only 85 women (27.2%) were included and selection bias existed. Fourth, the lack of tissue samples and hemodynamic data limited our ability to describe the relative influence of genetic factors on flow disturbances. Finally, the diagnosis of a raphe may not always be clear in surgical settings and from imaging modalities such as CT. When small or subtle raphes are accompanied by calcifications or other changes in valve structure, it is sometimes difficult to determine the presence or absence of raphes during CT imaging and surgery. Further prospective, multi-institutional studies with larger sample sizes are warranted.

## 5. Conclusions

In conclusion, the presence of a raphe was significantly associated with the type of aortopathy and degree of valve dysfunction. Furthermore, it has been identified as an independent determinant of AR.

## Figures and Tables

**Table 1:** Baseline and echocardiographic characteristics of patients with BAV–raphe+ vs. BAV–raphe−.

	Raphe+ (*n* = 185)	Raphe− (*n* = 127)	*p*-Value
Age	52.2 ± 14.5	53.4 ± 14.1	0.444
Sex (M)	156 (84.3)	71 (55.9)	<0.001
BSA	1.8 ± 0.2	1.7 ± 0.2	0.001
COPD	10 (5.4)	3 (2.3)	0.186
HTN	51 (27.6)	46 (36.2)	2.632
DM	14 (7.6)	9 (7.1)	0.873
Hyperlipidemia	35 (18.9)	17 (13.4)	0.198
Smoking	80 (43.2)	37 (29.1)	0.011
CAD	10 (7.9)	19 (10.3)	0.474
Coronary calcium score, median (range)	102.5 ± 341.5	68.6 ± 206.2	0.276
Valve calcium score, median	1523.8 ± 2333.9	2025.6 ± 2345.1	0.064
Aorta diameter (mm)			
Sinus of Valsalva	40.2 ± 6.4	38.7 ± 5.6	0.033
Tubular portion	42.1 ± 7	44.3 ± 8.3	0.015
Sinus of Valsalva/BSA	23 ± 3.7	23.1 ± 3.1	0.808
Tubular portion/BSA	24.2 ± 5	26.6 ± 5.6	<0.001
Aorta type			0.021
Normal	27 (14.6)	12 (9.5)	
Type 1	25 (13.5)	8 (6.3)	
Type 2	30 (16.2)	15 (11.8)	
Type 3	103 (55.7)	92 (72.4)	
Aortopathy	131 (70.8)	100 (78.7)	0.117
Aortic aneurysm	38 (20.5)	34 (26.8)	0.199
Aortic valve dysfunction			<0.001
Normal	4 (2.2)	15 (11.8)	
AR	91 (49.2)	13 (10.2)	
AS	33 (17.8)	64 (50.4)	
ASR	57 (30.8)	35 (27.6)	
AS severity			<0.001
None	95 (51.4)	28 (22.1)	
Mild	10 (5.4)	8 (6.3)	
Moderate	15 (8.1)	13 (10.2)	
Severe	65 (35.1)	78 (61.4)	
AR severity			<0.001
None	38 (20.5)	79 (62.2)	
Mild	64 (34.6)	31 (24.4)	
Moderate	34 (18.4)	10 (7.9)	
Severe	49 (26.5)	7 (5.5)	
Valve dysfunction dominance			<0.001
Dominant AS	70 (37.8)	82 (64.6)	
Dominant AR	73 (39.5)	8 (6.3)	
Balanced ASR	10 (5.4)	9 (7.1)	
None	32 (17.3)	28 (22.0)	
ECHO			
EDV (mL)	164.8 ± 81.1	124.6 ± 52.2	<0.001
ESV (mL)	65.4 ± 48.5	46.8 ± 36.1	<0.001
EF (%)	62.7 ± 11.8	63.6 ± 12.6	0.505
Valve OP	145 (78.4)	95 (74.8)	0.462
Aorta OP	66 (35.7)	55 (43.3)	0.174

AR = aortic regurgitation, AS = aortic stenosis, ASR = aortic stenosis and regurgitation, BSA = body surface area, CAD = coronary artery disease, COPD = chronic obstructive pulmonary disease, DM = diabetes mellitus, EDV = end-diastolic volume, ESV = end-systolic volume, EF = ejection fraction, HTN = hypertension, OP = operation, Type 1 = isolated dilation of the ascending aorta root, Type 2 = middle ascending dilatation, Type 3 = combined dilation of the ascending aorta and aortic root.

**Table 2:** Univariable and multivariable regression analyses to evaluate the association between raphe and aortic stenosis.

	Aortic Stenosis			
	Univariable		Multivariable	
Characteristics	Odds Ratio 95% CI	*p*-Value	Odds Ratio 95% CI	*p*-Value
Sex (male 1, female 0)	0.2 (0.1–0.4)	<0.001	0.2 (0.1–0.4)	<0.001
Age	1.1 (1.1–1.1)	<0.001	1.0 (1.0–1.0)	0.398
Aortic valve Ca score	1.0 (1.0–1.0)	<0.001	1.0 (1.0–1.0)	<0.001
EDV	1.0 (1.0–1.0)	<0.001	1.0 (1.0–1.0)	0.021
ESV	1.0 (1.0–1.0)	<0.001	1.0 (1.0–1.1)	0.701
EF	1.0 (1.0–1.1)	0.005	1.0 (1.0–1.1)	0.795
CAD	2.8 (1.2–6.8)	0.021	0.5 (0.1–3.1)	0.423
Hyperlipidemia	1.3 (0.7–2.3)	0.446	/	/
DM	1.6 (0.7–3.9)	0.301	/	/
Aortopathy	1.5 (0.9–2.6)	0.098	/	/
HTN	1.3 (0.8–2.1)	0.346	/	/
Raphe	0.3 (0.2–0.5)	<0.001	0.6 (0.3–1.5)	0.279

CAD = coronary artery disease, DM = diabetes mellitus, EDV = end-diastolic volume, ESV = end-systolic volume, EF = ejection fraction, HTN = hypertension.

**Table 3:** Univariable and multivariable regression analyses to evaluate the association between raphe and aortic regurgitation.

	Aortic Regurgitation			
	Univariable		Multivariable	
Characteristic	Odds Ratio 95% CI	*p*-Value	Odds Ratio 95% CI	*p*-Value
Sex (male 1, female 0)	7.7 (3.4–17.5)	<0.001	1.3 (0.4–4.3)	0.726
Age	1.0 (1.0–1.0)	<0.001	1.0 (0.9–1.0)	0.003
Aortic valve Ca score	1.0 (1.0–1.0)	<0.001	1.0 (1.0–1.0)	0.761
EDV	1.0 (1.0–1.1)	<0.001	1.1 (1.0–1.1)	<0.001
ESV	1.0 (1.0–1.1)	<0.001	1.0 (0.9–1.0)	0.025
EF	1.0 (0.9–1.0)	<0.001	1.0 (0.9–1.0)	0.142
CAD	0.1 (0.0–0.6)	0.008	0.3 (0.0–2.1)	0.215
Hyperlipidemia	0.6 (0.3–1.2)	0.132	/	/
DM	0.2 (0.0–0.8)	0.025	1.6 (0.2–14.7)	0.678
Aortopathy	1.0 (0.6–1.7)	0.992	/	/
HTN	0.6 (0.3–1.0)	0.035	0.9 (0.3–2.31)	0.756
Raphe	5.3 (2.9–9.5)	<0.001	3.7 (1.4–9.6)	0.007

CAD = coronary artery disease, DM = diabetes mellitus, EDV = end-diastolic volume, ESV = end-systolic volume, EF = ejection fraction, HTN = hypertension.

## Data Availability

The data that support the findings of this study are available as part of this article and no additional source data are required.
